# Association of serum fibroblast growth factor 19 levels with arteriosclerosis parameters assessed by arterial stiffness and atherogenic index of plasma in patients with type 2 diabetes

**DOI:** 10.1186/s13098-020-00552-0

**Published:** 2020-05-20

**Authors:** Wang-shu Liu, Meng-jie Tang, Tian-li Xu, Jian-bin Su, Xue-qin Wang, Feng Xu, Dong-mei Zhang, Qing Zhu, Jie Cao, Hong Wang

**Affiliations:** 1 Department of Endocrinology, Affiliated Hospital 2 of Nantong, University and First People’s Hospital of Nantong City, No. 6 North Hai‑er‑xiang Road, Nantong, 226001 China; 2 Medical College of Nantong University, No. 19 Qi-xiu Road, Nantong, 226001 China; 3 Medical Research Center, Affiliated Hospital 2 of Nantong University and First People’s Hospital of Nantong City, No. 6 North Hai‑er‑xiang Road, Nantong, 226001 China

**Keywords:** Type 2 diabetes, FGF19, baPWV, Atherogenic index of plasma

## Abstract

**Background:**

The role of serum fibroblast growth factor 19 (FGF19) in arteriosclerosis is not well known. In the present study, we aimed to explore whether serum FGF19 levels were related to arteriosclerosis parameters, including arterial stiffness and atherogenic index of plasma (AIP), in patients with type 2 diabetes (T2D).

**Methods:**

A total of 200 patients with type 2 diabetes and 50 healthy controls were recruited for this study from Apr 2017 to Oct 2018. Serum FGF19 levels, arterial stiffness assessed by brachial ankle pulse wave velocity (baPWV), and AIP assessed by the triglyceride to high-density lipoprotein cholesterol (TG/HDL-c) ratio were measured in those subjects. In addition, other relevant clinical data were also collected.

**Results:**

Serum FGF19 levels in T2D patients were significantly lower than those in healthy controls (*p *< 0.05). The arteriosclerosis parameters, including baPWV and AIP, significantly decreased across ascending tertiles of serum FGF19 levels (all *p for trend *< 0.001). Moreover, the baPWV and AIP were all inversely correlated with serum FGF19 levels (*r *= − 0.351 and − 0.303, respectively, *p *< 0.001). Furthermore, after adjusting for other clinical covariates by multiple linear regression analyses, the serum FGF19 levels were independently associated with baPWV (*β *= − 0.20, *t *= − 2.23, *p *= 0.029) and AIP (*β *= − 0.28, *t *= − 2.66, *p *= 0.010).

**Conclusions:**

The serum FGF19 levels were independently and inversely associated with baPWV and AIP, which indicate that serum FGF19 may have a protective role in atherosclerosis in patients with T2D.

## Background

Cardiovascular disease (CAD) is a complication with high morbidity and mortality in patients with type 2 diabetes (T2D) [1]. Serum lipid deposition, leucocyte infiltration, and intimal thickening are three main steps in the development of atherosclerosis [2]. Dyslipidaemia has been considered a mediator and marker of CAD.

Arterial stiffness, as noninvasively measured by brachial-ankle pulse wave velocity (baPWV), is considered to be an independent predictor of CAD. Previous studies have reported that baPWV was closely correlated with the incidence of CAD and CAD-related mortality [3, 4]. In addition, arterial stiffness is also a consequence of T2D and shared with the T2D in the pathogenesis of diabetic vascular complications [5, 6]. BaPWV was also closely associated with glycosylated haemoglobin A1c (HbA1c), regardless of diabetes status [7]. A growing body of evidence has shown that the atherogenic index of plasma (AIP) is an important indicator of atherosclerosis and CAD [8–10]. As a new comprehensive lipid index, AIP is calculated from the plasma triglyceride (TG) value divided by the high-density lipoprotein cholesterol (HDL-c) value [11]. The AIP levels in T2D patients were significantly higher than those in healthy controls and may be account for the distribution of body fat and visceral fat area (VFA) [12].

Fibroblast growth factor 19 (FGF19), an endocrine hormone, is a member of the fibroblast growth factor (FGF) family. FGF19 is mainly produced in the intestinal epithelium and is expressed and functions in target tissues via FGF receptors (FGFR1, 2, 3, and 4) and β-Klotho. The main function of FGF19 is to regulate the synthesis of bile acid (BA) by acting on cholesterol 7α-hydroxylase (CYP7A1) [13, 14]. As shown in our previous study, FGF19 participates in maintaining the balance of glucose and may protect against the dysfunction of β-cells [15]. The serum FGF19 levels in CAD subjects are lower than those in subjects without CAD and are correlated with the presence and severity of CAD [16]. However, few studies revealed the associations of FGF19 with arteriosclerosis indices, such as baPWV and AIP.

The aim of the present study was to investigate whether serum FGF19 levels were related to the arteriosclerosis parameters, including baPWV and AIP, in patients with type 2 diabetes. We found that serum FGF19 may have a protective role in atherosclerosis in patients with T2D.

## Methods

### Study design and population

We recruited 200 patients with type 2 diabetes who visited and were followed up in the Endocrinology Department at Affiliated Hospital 2 of Nantong University from April 2017 to October 2018, and 50 healthy controls were selected from the Department of Physical Examination Center during the same period. The inclusion criteria for T2D patients were age from 20 to 75 years and a diagnosis of T2D [17]. The inclusion criteria for controls were age from 20 to 75 years and having a fasting plasma glucose (FPG) level < 6.1 mmol/L and a 2-h plasma glucose (2hPG) level < 7.8 mmol/L [17]. Subjects who had type 1 diabetes, hyperthyroidism or hypothyroidism, severe hepatic disease such as chronic viral hepatitis, chronic renal insufficiency with estimated glomerular filtration rate < 90 ml/min/1.73 m^2^, previous cancer, acute diabetic complications such as diabetic ketoacidosis, and current treatment with systemic corticosteroids were also excluded. The brachial-ankle pulse wave velocity (baPWV) was determined by a non-invasive vascular screening device (BP-203RPE III device, Omron Healthcare, Kyoto, Japan) while the patient was in the supine position and after a rest of at least 5 min. All participants provided informed consent, and the study protocol was approved by the Ethics Committee of Affiliated Hospital 2 of Nantong University and First People’s Hospital of Nantong City.

### Anthropometric measures and calculation

All participants finished a questionnaire collecting demographic and anthropometric data under the supervision of experienced investigators after fasting for 8 h. Hypertension was tested at least 3 times and was defined as a systolic arterial blood pressure ≥ 140 mmHg and/or diastolic blood pressure ≥ 90 mmHg after at least 0.5 h of rest. Hepatic steatosis on ultrasound was used to establish the diagnosis of non-alcoholic fatty liver disease (NAFLD). The calculation of the homeostatic model assessment for insulin sensitivity (IS_HOMA-cp_) [18], Body mass index (BMI), AIP and NAFLD fibrosis score (NFS) was as follows: IS_HOMA-cp_ = 22.5/ (glucose × C-peptide in fasting status); BMI = the weight/the height squared; AIP = TG/HDL-c; NFS = − 1.675 + 0.037 × age (years) + 0.094 × BMI (kg/m^2^) + 1.13 × IFG/diabetes (yes = 1, no = 0) + 0.99 × AST/ALT ratio– 0.013 × platelet (× 10^9^/l) − 0.66 × albumin (g/dl) [15].

### Serum biochemical indicators

Peripheral blood samples were collected after > 12 h of fasting. TG was measured using colorimetry, total cholesterol (TC) was measured with the cholesterol oxidase method, low-density lipoprotein cholesterol (LDL-c) was determined with the selective melt method and HDL-c was measured using the modified enzyme method. All levels were determined by an automated biochemical instrument (Model 7600, Hitachi). Ionic exchange HPLC (IE-HPLC) in the D-10 haemoglobin analysis system (Bio-Rad) was used to determine the HbA1c level. Insulin levels, fasting blood glucose concentrations and 2-h blood glucose concentrations were measured by laboratory procedures [16, 17]. All blood samples were stored at − 80 °C. Serum FGF19 levels were measured by sandwich ELISA (FGF19 Quantikine^®^ DF1900; R&D Systems, Minneapolis, MN, USA). The intra- and inter-assay coefficients of variation were 4.3% and 5.6%, respectively.

### Statistical analysis

SPSS 25.0 (Inc., Chicago, IL) statistical software was used. Normally distributed data are expressed as the mean ± SD. The Kruskal–Wallis test was performed to assess the distributions of the variables. If the distributions of the data were skewed, a natural logarithm transformation (ln) was applied to achieve a normal distribution for further analysis, such as lnFGF19 and lnAIP. The differences in continuous variables between the FGF19 tertiles were compared by one-way analysis of variance (ANOVA), and the categorical variables between the three groups were compared by the Chi square test. Spearman’s bivariate correlation analysis was used to analyse the correlations of lnFGF19 with baPWV and lnFGF19 with lnAIP. Considering that age, gender, duration, BMI, hypertension, diabetic treatment and NAFLD may affect the values of baPWV and AIP, two partial correlation analyses were conducted to explore the associations of lnFGF19 with baPWV and lnFGF19 with lnAIP, adjusting for age, gender, duration, BMI, hypertension, diabetic treatment and NAFLD. Furthermore, two multiple stepwise linear regression analyses were used to explore the independent factors related to FGF19 levels for baPWV and to FGF19 levels for AIP. A *p* value < 0.05 was defined as significant.

## Results

### Basic characteristics

The characteristics of the participants are shown in Table 1. Compared with healthy controls, T2D patients were older and had higher BMI, systolic pressure (SBP), diastolic blood pressure (DBP), fasting plasma glucose (FPG) and 2-h postprandial blood glucose (2hPG) (all *p* < 0.01). The FGF19 concentrations were significantly lower in the T2D group than the control group. The mean FGF19 levels for the whole T2D group were 138.18 (66.46–181.31), and the tertiles were T1 (< 82.6), T2 (82.6–149.6), and T3 (> 149.6). According to the examination results, prominent differences were found for AIP and baPWV from T1 to T3 (all *p for trend* < 0.001). Moreover, from T1 to T3, the number of T2D patients with hypertension decreased significantly (*p for trend* < 0.05), but no significant difference was found in the number of T2D patients with NAFLD and the NFS values. The mean NFS for T2D participants was − 23.71 ± 2.65, which indicates that for most T2D patients with NAFLD, the negative predictive value for fibrosis was 93% [19].

**Table 1:** Characteristics of the study participants

Variable	Controls	T2D				*p* for trend
		Total	T1	T2	T3	
FGF19 (pg/mL)	232.3 (122.8–280.8)	138.2 (66.5–181.3)**	< 82.6	82.6–149.6	> 149.6	
N (male %)	50 (19.6)	200 (60.5)**	67 (62.7)	67 (53.7)	66 (65.2)	0.776
Diabetes duration (months)	N	49.23 (1.0–72.0)	51.38 (2.0–96.0)	61.22 (1.0–96.0)	34.87 (1.0–36.25)	0.185
Age (years)	35.3 ± 10.9	52.4 ± 10.7**	52.1 ± 11.2	52.3 ± 10.2	53.0 ± 10.8	0.626
BMI (kg/m2)	22.5 ± 2.7	25.8 ± 3.6**	26.3 ± 3.5	26.0 ± 3.1	25.2 ± 4.1	0.061
SBP (mmHg)	109.8 ± 7.1	136.2 ± 16.4**	134.6 ± 16.8	136.7 ± 15.0	137.5 ± 17.4	0.300
DBP (mmHg)	76.0 ± 9.5	81.9 ± 11.2**	80.8 ± 10.8	81.7 ± 9.9	83.3 ± 12.7	0.211
Antidiabetic treatment						
Lifestyle intervention alone, n (%)	N	2 (1.0)	0	1 (1.5)	1 (1.5)	0.252
Insulin treatments, n (%)	N	136 (68.0)	43 (64.2)	49 (73.1)	44 (66.7)	0.250
Insulin-secretagogues, n (%)	N	87 (43.5)	27 (40.3)	32 (47.8)	28 (42.4)	0.290
Insulin-sensitisers, n (%)	N	24 (12.0)	6 (9.0)	10 (14.9)	8 (12.1)	0.511
Hypertension, n (%)	N	89 (44.5)	33 (49.3)	27 (40.3)	29 (43.9)	0.047^#^
Statins, n (%)	N	17 (8.5)	10 (14.9)	3 (4.5)	4 (6.1)	0.711
NAFLD, n (%)	N	89 (44.5)	35 (52.2)	25 (37.3)	29 (43.9)	0.264
NFS	N	− 23.71 ± 2.65	− 24.25 ± 2.61	− 23.20 ± 2.69	− 23.71 ± 2.59	0.264
FPG (mmol/L)	4.63 ± 0.20	6.12 ± 1.72**	6.04 ± 1.77	6.18 ± 1.82	6.13 ± 1.57	0.766
2hPG (mmol/L)	9.24 ± 2.28	16.78 ± 3.75**	16.39 ± 3.95	17.40 ± 3.28	16.54 ± 3.98	0.833
HbA1c (%)	N	9.46 ± 2.29	9.41 ± 2.22	9.91 ± 2.54	9.01 ± 2.02	0.346
IS_HOMA-cp_	N	10.06 (2.72–8.65)	9.91 (2.81–9.16)	12.86 (2.72–10.15)	7.33 (2.37–7.64)	0.720
UA (μmol/L)	N	314.36 (250.0–377.0)	323.76 (251.0–404.0)	309.7 (248.3–357.5)	309.5 (242.5–380.5)	0.434
TC (mmol/L)	4.31 ± 0.80	4.44 ± 0.82	4.42 ± 0.86	4.61 ± 0.86	4.29 ± 0.71	0.377
TG (mmol/L)	1.22 (0.60–1.54)	2.11 (1.32–2.67)**	2.45 (1.58–3.07)	2.12 (1.20–2.74)	1.76 (1.18–2.17)	0.001^###^
HDL-c (mmol/L)	1.34 ± 0.17	1.12 ± 0.29**	1.06 ± 0.26	1.13 ± 0.23	1.17 ± 0.35	0.025^#^
LDL-c (mmol/L)	2.54 ± 0.56	2.82 ± 0.70*	2.82 ± 0.70	2.96 ± 0.70	2.67 ± 0.67	0.198
LPa (mg/L)	N	186.5 (45.0–243.0)	178.7 (35.0–264.0)	171.7 (45.0–230.0)	223.2 (49.0–245.0)	0.445
InAIP	− 0.13 ± 0.30	0.23 ± 0.25**	0.33 ± 0.23	0.23 ± 0.27	0.15 ± 0.21	0.001^###^
baPWV (cm/s)	N	1879.8 ± 355.2	2029.6 ± 330.3	1870.0 ± 329.4	1737.7 ± 348.9	0.001^###^

Categorical variables are frequency (percentage), normally distributed values in the table are mean ± SD and non-normally distributed values are median (25 and 75% interquartiles)

*FGF19* fibroblast growth factor 19, *BMI* body mass index, *SBP/DBP* systolic/diastolic blood pressure, *NAFLD* non-alcoholic fatty liver disease, *FPG* fasting plasma glucose, *2hPG* 2-h postprandial blood glucose, *HbA1c* glycosylated hemoglobin A1c, *IS*_*HOMA-cp*_ homeostasis model assessment for insulin sensitivity calculated using glucose and C-peptide in fasting status, *UA* uric acid, *TC* total cholesterol, *TG* triglyceride, *HDL-c* high density lipoprotein cholesterol, *LDL-c* low density lipoprotein cholesterol, *LPa* lipoprotein a, *AIP* atherogenic index of plasma, *baPWV* brachial-ankle pulse wave velocity, *NFS* NAFLD fibrosis score

p values for continuous variables and categorical variables were determined by ANOVA and the Chi squared test, respectively. ^#^ p < 0.05, ^##^ p < 0.01, ^###^ p < 0.001

* P < 0.05, ** P < 0.01, the comparison of T2D with Controls

### Relationships between lnFGF19 and arteriosclerosis parameters

**Fig. 1:**
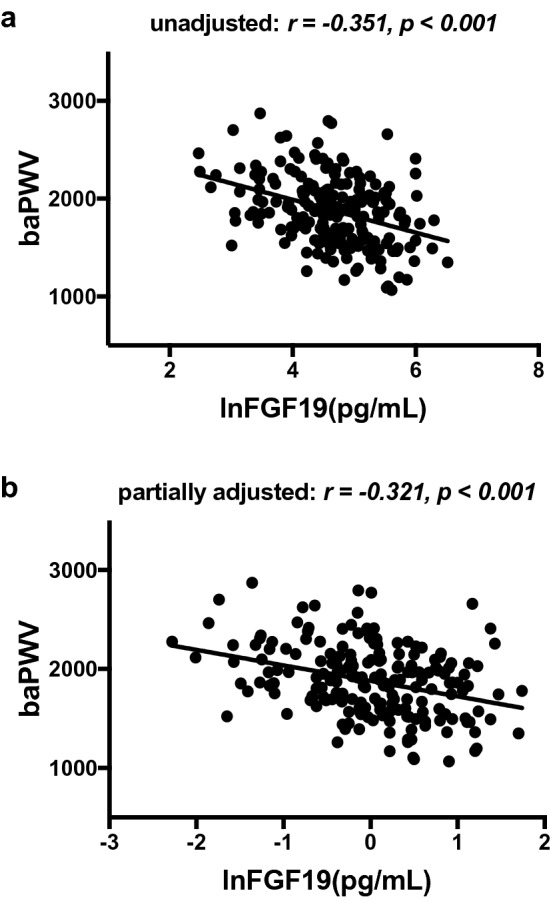
The relationship between lnFGF19 and baPWV in patients with T2D (**a** unadjusted; **b** partially adjusted for age, gender, duration, BMI, hypertension, diabetic treatments, NAFLD)

**Fig. 2:**
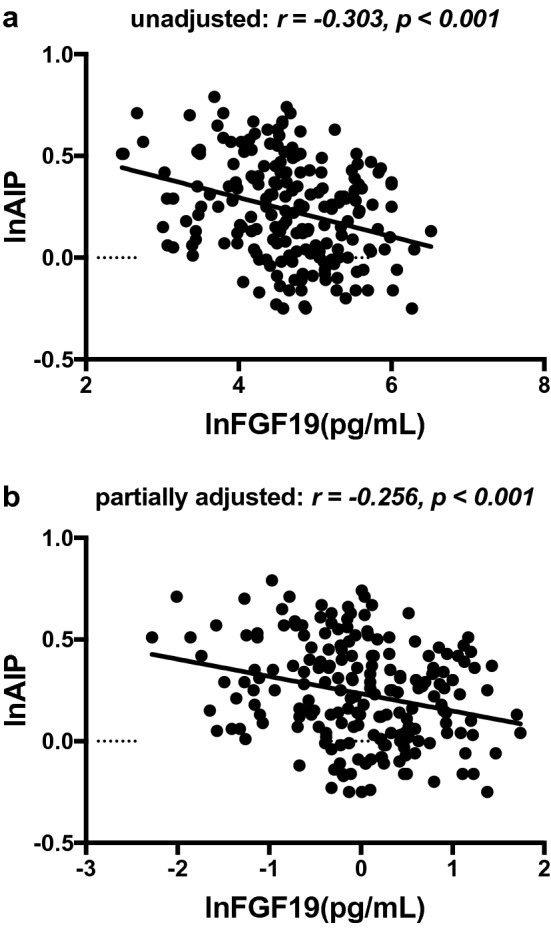
The relationship between lnFGF19 and lnAIP in patients with T2D (**a** unadjusted; **b** partially adjusted for age, gender, duration, BMI, hypertension, diabetic treatments, NAFLD)

Spearman’s bivariate correlation analysis was conducted to assess the relationships between lnFGF19 levels and arteriosclerosis parameters in the T2D group. As shown in Figs. 1, 2, the serum lnFGF19 levels were significantly and negatively related to baPWV and lnAIP (*r *= − 0.351, *p *< 0.001; *r *= − 0.321, *p *< 0.001, respectively). Interestingly, the associations all still existed after adjusting for age, gender, duration, BMI, hypertension, diabetic treatment and NAFLD (*r* = − 0.321, *p* < 0.001; r = − 0.256, *p* < 0.001, respectively).

### Multiple linear regression models displayed independent associations of lnFGF19 with arteriosclerosis parameters

Table 2 shows the associations of lnFGF19 with arteriosclerosis parameters (baPWV and AIP) based on multiple linear regression analyses. In the basal unadjusted model 0, lnFGF19 was significantly associated with baPWV (*β *=− 0.37, *t *= − 5.57, *p *< 0.001, adjusted *R*^*2*^= 0.135) and lnAIP (*β *= − 0.30, *t *= − 4.46, *p *< 0.001, adjusted *R*^*2*^= 0.091). After gradually adding the other clinical covariates in each model, we observed a gradual increase in the adjusted *R*^*2*^. In the fully adjusted model 3, lnFGF19 was still independently associated with baPWV (*β *= − 0.20, *t *= − 2.23, *p *=0.029, adjusted *R*^*2*^= 0.599) and lnAIP (*β *=− 0.28, *t *= − 2.26, *p *= 0.010, adjusted *R*^*2*^= 0.491). As a result, the serum FGF19 levels were independently and inversely associated with baPWV and AIP in patients with T2D.

**Table 2:** Multiple linear regression models displaying adjusted estimates for lnFGF19 for outcomes of arteriosclerosis parameters adjusted for the other clinical covariates in each model in patients with T2D

Models	B (95% CI)	*β*	*t*	*p*	*R*^*2*^ for model
baPWV					
Model 0: unadjusted	− 167.6 (− 227.0 to − 108.3)	− 0.37	− 5.57	< 0.001	0.135
Model 1: age, sex, BMI and duration	− 161.8 (− 218.7 to − 104.9)	− 0.36	− 5.61	< 0.001	0.241
Model 2: Model 1 + SBP, DBP, hypertension, statins, NAFLD, NFS, UA, TC, TG, HDLC, LDLC, and Lpa	− 98.7 (− 171.5 to − 26.0)	− 0.22	− 2.70	0.008	0.587
Model 3: Model 2 + IS_HOMA-cp_, HbA1c and antidiabetic treatments	− 86.8 (− 164.5 to − 9.1)	− 0.20	− 2.23	0.029	0.599
lnAIP					
Model 0: unadjusted	− 0.22 (− 0.32 to − 0.12)	− 0.30	− 4.46	< 0.001	0.091
Model 1: age, gender, BMI and diabetic duration	− 0.21 (− 0.30 to − 0.11)	− 0.28	− 4.19	< 0.001	0.145
Model 2: Model 1 + SBP, DBP, hypertension, statins, NAFLD, NFS, UA, TC, LDLC and Lpa	− 0.21 (− 0.35 to − 0.07)	− 0.29	− 3.03	0.003	0.342
Model 3: Model 2 + IS_HOMA-cp_, HbA1c and antidiabetic treatments	− 0.21 (− 0.36 to − 0.05)	− 0.28	− 2.66	0.010	0.491

## Discussion

We conducted an observational study to compare the serum FGF19 levels between the patients with T2D and healthy controls and to analyse the association of serum FGF19 levels with baPWV and FGF19 levels with AIP in patients with T2D. The main findings of our study are as follows: first, serum FGF19 levels of T2D patients were significantly lower than those in healthy controls; second, arteriosclerosis parameters, including baPWV and AIP, significantly decreased across ascending tertiles of serum FGF19 levels in patients with T2D; and third, after adjusting for other clinical covariates, the serum FGF19 levels were independently and inversely associated with baPWV in patients with T2D.

Evidence from previous studies has shown that FGF19 participates in the synthesis of BA, the balance of glucose metabolism, and the reduction of weight in mice [20–23]. In accordance with our data, J. Zhang et al. showed that serum FGF19 levels were significantly lower in normal glucose tolerance (NGT) subjects than in isolated-impaired glucose tolerance (I-IGT) subjects and isolated-impaired fasting glucose (I-IFG) participants based on glucose effectiveness (GE) and hepatic glucose production (HGP) [24]. Meanwhile, in T2D patients with MetS, serum FGF19 levels were significantly lower than they were in other T2D patients. Moreover, FGF19 levels were significantly negatively related to AIP and TG in T2D patients with MetS [25]. In a study of 315 Chinese patients with suspected or established CAD, the serum FGF19 level was an independent predictor of the Gensini score, which represents the presence and severity of CAD [16]. In our study, the FGF19 levels in the T2D group decreased significantly compared with those in the control group. In the T2D group, lnFGF19 levels were negatively related to baPWV and AIP values.

Arterial stiffness was considered to be an independent predictor of atherosclerotic diseases and can be measured by baPWV, a simple, noninvasive and convenient tool [26, 27]. Previous studies revealed that baPWV was broadly used and generally accepted in China [28]. BaPWV was associated with blood pressure, diabetes status [29], HbA1c [7], inflammation [30] and obesity [31]. We considered baPWV as a risk factor for CVD and investigated its association with serum FGF19 levels. Finally, our study found that in the fully adjusted model 3, lnFGF19 was still independently associated with baPWV (*β *= − 0.20, *t *= − 2.23, *p *=0.029, adjusted *R*^*2*^= 0.599).

AIP is a main marker for the presence of atherosclerosis and CAD [32, 33]. In postmenopausal women with CAD, the values of AIP were higher than those in the control group. After multivariate logistic regression analysis, AIP was shown to be independently related to CAD, which indicated that AIP was a significant marker for the incidence of CAD [34]. Gaojun Cai et al. found that in the Han Chinese population, AIP was a predictor for the incidence of CAD [33]. The patients with diabetic neuropathy and MetS had significantly higher AIP levels than their counterparts [35]. In our study, the levels of AIP in the T2D group were higher than those in the controls, and AIP was independently associated with the serum FGF19 levels in T2D patients. In agreement with our study, P. Song et al. revealed that the AIP was obviously higher in T2D participants than in non-T2D participants [12]. However, more research should be conducted to analyse the relationship between FGF19 levels and arterial lesions.

Several possible mechanisms may explain the link between FGF19 levels and atherosclerosis in T2D patients. FGF19 regulates glucolipid homeostasis and nutrient metabolism via the FGFR4-β-Klotho complex. In rodent studies, FXR-/- mice showed a pro-atherogenetic lipoprotein profile and defects in the formation of any detectable plaques associated with a high-fat (HF) diet. FXR agonists protect against the formation of aortic plaques in murine models that have a pro-atherogenetic lipoprotein profile and accelerated atherosclerosis [36]. In hepatic FXR-knockout and FXR-knockdown mice, the reconstitution of FXR expression upregulated the transport of cholesterol. Consistent with its role of phosphorylating FXR, the nonreceptor tyrosine kinase Src regulated the formation of cholesterol and ameliorated arterial lesions. Therefore, the phosphorylation of hepatic FXR induced Src via FGF15/19 and then played a role in the balance of cholesterol homeostasis, preventing the formation of atherosclerosis [37]. In transgenic human apolipoprotein (a) (APOA) mice (tg-APO mice), the FGF19 influence APOA biosynthesis by attenuating transfection of primary hepatocytes with siRNA against the FGFR4 [38]. Moreover, Mei Zhou et al. revealed that NGM282, an FGF19 analogue, regulated cholesterol in mice by activating MEK1 and reduced atherosclerosis in Apoe-/- mice with dyslipidaemia. Furthermore, the HDL-c levels of healthy volunteers improved after the administration of NGM282 for a week [39]. Therefore, our research revealed that baPWV and AIP were all independently associated with FGF19 levels after adjusting for other clinical covariates via multiple linear regression analysis, which indicates that decreased FGF19 may be accompanied by several unfavourable metabolic alterations and can be implicated in increased arterial stiffness.

This study had several limitations that should be addressed. First, this study had a cross-sectional design based on a small sample size and could not explain any causal connection between the decreased serum FGF19 level and increased risk of atherosclerosis and CAD as assessed by baPWV and AIP. Second, our research was a single-centre study conducted among Chinese participants, and the generalizability of our data needs to be assessed. Third, the influence of diabetic and hypertensive treatment on the formation of atherosclerosis is unknown, and the influence of medication history on baPWV and AIP values has not been determined. However, lnFGF19 levels were closely related to baPWV and AIP, even after adjusting for age, gender, duration, BMI, hypertension, diabetic treatment and NAFLD. Meanwhile, baPWV was not measured among controls, but the present research showed that diabetes was significantly correlated with an increased risk of baPWV compared with normal glucose [29]. Moreover, other confounding factors affecting baPWV, such as alcohol consumption, smoking status and health behaviour, were not completely assessed at the beginning of this study. Finally, the relationships between FGF19 levels and CAD measured by other vascular markers, such as carotid intima-media thickness (CIMT) or flow-mediated dilatation (FMD), were not assessed. All limitations were fully considered when selecting the appropriate statistical approach. Therefore, larger, prospective follow-up studies need to be conducted to better investigate the correlation between FGF19 levels and the risk of CAD in T2D patients.

## Conclusions

The serum FGF19 levels were independently and inversely associated with baPWV and AIP, which indicate that serum FGF19 may have a protective role in atherosclerosis in patients with T2D.

## Data Availability

The current data are available to all interested researchers upon reasonable request. Requests for access to data should be made to the principal investigators of the study.

## Abbreviations

CAD: Cardiovascular disease
T2D: Type 2 diabetes
baPWV: Brachial-ankle pulse wave velocity
CN: Controls
BMI: Body mass index
SBP: Systolic blood pressure
DBP: Diastolic blood pressure
FPG: Fasting plasma glucose
2hPG: 2-hour postprandial blood glucose
HbA1c: Glycosylated haemoglobin A1c
BA: Bile acid
CYP7A1: Cholesterol 7α-hydroxylase
IS_HOMA-cp_: Homeostasis model assessment for insulin sensitivity calculated using glucose and C-peptide in fasting status
TC: Total cholesterol
TG: Triglyceride
HDL-c: High-density lipoprotein cholesterol
LDL-c: Low-density lipoprotein cholesterol
FGF19: Fibroblast growth factor 19
AIP: Atherogenic index of plasma
OGTT: Oral glucose tolerance test
NGT: Normal glucose tolerance
I-IGT: Isolated-impaired glucose tolerance
I-IFG: Isolated-impaired fasting glucose
GE: GLUCOSE effectiveness
HGP: Hepatic glucose production
MetS: Metabolic syndrome
HF: High-fat
NAFLD: Non-alcoholic fatty liver disease
NFS: NAFLD fibrosis score
CIMT: Carotid intima-media thickness
FMD: Flow-mediated dilatation
APOA: Apolipoprotein (a)
